# Mutations in ParC and GyrA of moxifloxacin-resistant and susceptible *Mycoplasma genitalium* strains

**DOI:** 10.1371/journal.pone.0198355

**Published:** 2018-06-08

**Authors:** Ryoichi Hamasuna, Phuong Thi Le, Satoshi Kutsuna, Keiichi Furubayashi, Masahiro Matsumoto, Norio Ohmagari, Naohiro Fujimoto, Tetsuro Matsumoto, Jorgen Skov Jensen

**Affiliations:** 1 Department of Urology, University of Occupational and Environmental Health, Kitakyushu, Japan; 2 Disease Control and Prevention Center, International Health Care Center, National Center for Global Health and Medicine, Tokyo, Japan; 3 Sonezaki Furubayashi Clinic, Osaka, Japan; 4 Research Unit for Reproductive Tract Microbiology, Statens Serum Institut, Copenhagen, Denmark; Miami University, UNITED STATES

## Abstract

Macrolide or fluoroquinolone-resistant *Mycoplasma genitalium* is spreading worldwide. We aimed to determine the influence of single nucleotide polymorphisms (SNPs) in the quinolone resistance determining regions (QRDR) of *parC* and *gyrA* in cultured *M*. *genitalium* strains. In addition, we examined the prevalence of macrolide- and fluoroquinolone resistance mediating mutations in specimens collected from Japanese male patients with urethritis in two time-periods between 2005–2009 and 2010–2017, respectively, by sequencing the QRDR of *parC* and *gyrA* and domain V of the 23S rRNA gene. The minimum inhibitory concentrations (MIC) of moxifloxacin, sitafloxacin, ciprofloxacin, levofloxacin, doxycycline, minocycline, azithromycin and clarithromycin were determined in 23 *M*. *genitalium* strains. Three cultured strains had elevated MICs for moxifloxacin at 16, 4 and 2 mg/L and had SNPs with the amino-acid change Ser83→Ile in ParC (*p*<0.001) and 3 kinds of SNPs with amino-acid changes Asp99→Asn, Gly93→Cys and Met95→Ile in GyrA, respectively. Among a total of 148 *M*. *genitalium* positive urine specimens, the prevalence of A2058G and A2059G SNPs in the 23S rRNA gene and any SNPs in ParC increased from 4.8% and 22.6% in 2005–2009 to 42.2% and 53.1% in 2010–2017, respectively. If *M*. *genitalium* is considered multi-drug resistant in clinical specimens carrying SNPs in the 23S rRNA gene and Ser83→Ile in ParC, the prevalence of multi-drug resistance is 12.5% in 2010–2017 in Japan. In conclusion, the SNP resulting in Ser83→Ile in ParC is closely related to moxifloxacin resistance even though other factors may also affect treatment outcomes by moxifloxacin. The prevalence of circulating multi-drug resistant *M*. *genitalium* strains with macrolide- and fluoroquinolone-resistance is dramatically increasing in Japan.

## Introduction

*Mycoplasma genitalium* is a pathogen causing male urethritis, female cervicitis and other sexually transmitted infection syndromes [1–3]. The treatment strategies for *M*. *genitalium* infections have been widely discussed [4]. Macrolide regimens, especially using azithromycin (AZM) were thought to be superior to tetracycline regimens for *M*. *genitalium* infections [5–7], and the AZM regimens have been widely used as the first line treatment for non-gonococcal urethritis in many countries. However, treatment failure by AZM regimens in *M*. *genitalium* urethritis in men has been reported [8], and AZM resistant *M*. *genitalium* strains were subsequently isolated [9]. This resistance was closely related to mutations in positions A2058 or A2059 of the 23S rRNA gene (*Escherichia coli* numbering) mainly to G (C or T is relatively rare). These two positions are in the active site for macrolide binding, and similar mutations have been observed among macrolide resistant *Mycoplasma pneumoniae* [10]. Several *M*. *genitalium* treatment-failures after AZM have been reported in various countries. The prevalence of macrolide resistance in *M*. *genitalium* has reached 50% in Australia [11], Denmark [12], and Japan [13, 14], and macrolide resistant *M*. *genitalium* has become a problem worldwide.

Fluoroquinolones act on the DNA gyrase and topoisomerase IV which are essential for cell survival and reproduction and have been used to treat *Chlamydia trachomatis* and mycoplasmas [15, 16]. While the *in vitro* and *in vivo* activities of ciprofloxacin (CIP) and levofloxacin (LVX) against *M*. *genitalium* infections were low [13, 17–19], moxifloxacin (MXF) or sitafloxacin (STFX) have been successful in eradicating *M*. *genitalium* treatment failures after AZM [8, 13, 20]. However, MXF or STFX treatment failures have been increasingly common [11, 13, 16, 20, 21], and Jensen *et al*. documented five *M*. *genitalium* strains with high MXF minimum inhibitory concentrations (MICs) [22]. Several reports have shown single nucleotide polymorphisms (SNPs) in the quinolone resistance determining regions (QRDR) of gyrase or topoisomerase genes detected from *M*. *genitalium* positive clinical specimens, similar to those found in other fluoroquinolone resistant bacteria [11, 13, 20, 21, 23, 24]. However, the correlation between SNPs in *gyrA* or *parC* and MXF or STFX resistance has not been clear, because of the lack of data from cultured strains of *M*. *genitalium*. In this study, we isolated two *M*. *genitalium* strains with high MICs to MXF and analyzed the relationships between MICs of fluoroquinolones and mutations in *gyrA* or *parC* genes of available *M*. *genitalium* strains. In addition, we examined the prevalence of SNPs in *M*. *genitalium* positive clinical specimens and showed the trends of antimicrobial resistance in Japan.

## Materials and methods

### *M*. *genitalium* strains

All *M*. *genitalium* strains were stored frozen at -80°C. G37^T^ and an early passage of the M30 were obtained from Statens Serum Institut, Research Unit for Reproductive Tract Microbiology, Copenhagen, Denmark [25]. The remaining 21 strains were primary isolates obtained from urethral swabs or urine sediment specimens from male patients in the same laboratory: M2282, M2300, M2321 and M2341 were isolated from Danish men [26]; M6090, M6151 and M6312 were from three consecutive specimens from a French man [25]; M6257, M6280, M6281, M6285, M6286, M6328, and M6489 were from Swedish men [18, 22]; M6282, M6283, M6284, M6287, IMC-1, OSSP35-2, and JMPP4 were from Japanese men [18, 27]. IMC-1, OSSP35-2 and JMPP4 were isolated in the laboratory of the Department of Urology, University of Occupational and Environmental Health, Kitakyushu, Japan in 2017. The methods for isolation of *M*. *genitalium* have been previously described [9, 26–28].

### Antimicrobial susceptibility testing

For 17 strains capable of growing in modified SP4 mycoplasma medium, MICs were determined by the modified broth dilution method as described by Hannan [29]. For the remaining six strains, (M6281, M6287, M6312, IMC-1, OSSP35-2, and JMPP4), not capable of growing in axenic medium, MICs were determined by the Vero cell-culture method with measurement of growth by quantitative TaqMan PCR as previously described [18, 28]. MIC values were defined as the lowest concentration of antimicrobials achieving >99% inhibition of growth compared with DNA loads of *M*. *genitalium* cultured in medium without antimicrobials. The MICs determined by the two different methods were comparable as shown previously [18, 19]. Antimicrobials included in the study were CIP, LVX, MXF, STFX, doxycycline (DOX), minocycline (MIN), AZM, and clarithromycin (CLR). STFX was provided by Daiichi Sankyo Co. Ltd, Tokyo, Japan and all other agents were purchased from Sigma-Aldrich, Tokyo, Japan.

### *M*. *genitalium* positive clinical specimens

A total of 148 *M*. *genitalium* positive first void urine specimens were collected from male patients in Japan between 2006 and 2017 before treatment. *M*. *genitalium* was detected by TaqMan^™^ realtime PCR [25]. During the period 2006–2009, 61 and 22 specimens were collected in clinical studies to isolate *M*. *genitalium* strains [18, 27, 30] and a clinical trial of gatifloxacin [15], respectively. During the period 2010–2017, 26 specimens were collected in a clinical trial for sitafloxacin [16] and 23 and 11 were collected in two clinical studies [30, 31]. In addition, four urine specimens sent from two Japanese clinics to the laboratory at the Department of Urology, University of Occupational and Environmental Health, Japan with the request for detection of *M*. *genitalium*. DNA was extracted from the urine specimens using the QIAamp DNA Mini Kit (QIAGEN, GmbH, Hilden, Germany) according to the manufacturer’s instruction. Clinical specimens were collected at the patients’ first visit at the medical facility.

### DNA sequencing

Macrolide resistance mediating mutations were detected from cultured strains and clinical specimens by sequencing of domain V of the 23S rRNA gene as previously described [9] using primers 23S-1992F (5'-CCATCTCTTGACTGTCTCGGCTAT) and 23S-2138R (5'-CCTACCTATTCTCTACATGGTGGTGTT) in a conventional PCR. The 147 bp amplicon was sequenced using BigDye Terminator v3.1 Cycle Sequence Kit (Applied Biosystems, Foster City, CA) on an ABI 3030x1 (Applied Biosystems).

The SNPs for fluoroquinolone resistance were detected from cultured strains and clinical specimens by sequencing the QRDR of the *gyrA* and *parC* genes as previously described [23]. Nucleotides 172–402 of *gyrA* were amplified using primers gyrA-F (5'-CGTCGTGTTCTTTATGGTGC) and gyrA-R (5'-ATAACGTTGTGCAGCAGGTC). Nucleotides 164–483 of *parC* were amplified using primers parC-F (5'-TGGGCTTAAAACCCACCACT) and parC-R (5'- CGGGTTTCTGTGTAACGCAT) [23]. The target amplicons were sequenced using the amplification primers.

### Statistical analysis

For analysis of the relation between antimicrobial susceptibility and mutations in *parC*, *gyrA*, or 23S rRNA gene, the MICs of *M*. *genitalium* strains were dichotomized at MICs ≥ 1 mg/L for quinolones and macrolides. The presence of mutations in the 23S rRNA gene or the QRDR of *parC* in relation to resistance was compared by Fisher’s exact test. P-values less than 0.05 were considered significant.

For analyzing the difference in prevalence of mutations in the 23S rRNA or QRDR of *gyrA* or *parC* among *M*. *genitalium* positive clinical specimens collected in 2005–2009 and 2010–2017, Fisher’s exact test was used.

### Ethics

This study was approved by the Human and Animal Ethics review committee of the university of Occupational and Environmental Health, Japan (no. H29-104). All personal data were anonymized at the collection of the urine specimens.

## Results

### MICs of selected antimicrobials for 23 strains and the correlation with SNPs in the QRDR of *gyrA* or *parC* or the 23S rRNA gene

The MICs and the SNPs with corresponding amino acid changes in the QRDR of *gyrA* or *parC* and the 23S rRNA genes are shown in Table 1. Four strains (M6257, M6489, IMC-1 and OSSP35-2) with MIC >16 mg/L were resistant to macrolides. These strains had A2058G and A2059G SNPs in the 23S rRNA gene. The macrolide MICs of the other strains were low (range: 0.0005–0.002 mg/L for AZM; 0.001–0.008 mg/L for CLR) corresponding with the wild-type sequence found in positions 2058 and 2059 in domain V of the 23S rRNA gene (p<0.001).

**Table 1:** Correlation between MICs for selected antimicrobials and SNPs in domain V of the 23S rRNA gene and the QRDRs of GyrA (NA: 127–402, AA: 57–134) and ParC (NA: 164–483, AA: 55–161) among 23 *M*. *genitalium* isolates. (Numbering of amino acids in *M*. *genitalium* numbering; *E*. *coli* numbering in brackets).

Strain name	Year of sampling	Country	Mutations of DNA/amino acid			Antimicrobials (mg/L)							
			QRDR		23S rRNA	Fluoroquinolone				Tetracycline		Macrolide	
			GyrA	ParC	-	CIP	LVX	MXF	STFX	DOX	MIN	AZM	CLR
**G37**^**T**^	1980	UK	-	-	-	**8**	**2**	0.06	0.125	0.5	0.5	0.002	0.004
**M30**	1980	UK	-	-	-	**4**	**1**	0.06	0.125	0.5	0.25	0.002	0.008
**M2282**	1991	DK	-	-	-	**4**	**1**	0.06	0.125	0.5	0.25	0.002	0.008
**M2300**	1991	DK	-	-	-	**8**	**2**	0.125	0.25	0.125	0.25	0.001	0.004
**M2321**	1991	DK	-	-	-	**4**	**2**	0.125	0.125	0.5	0.25	0.0005	0.001
**M2341**	1991	DK	T378C	Pro62(59)→Ser C234T	-	0.25	0.25	0.06	0.015	0.5	0.25	0.002	0.008
**M6090**	1994	FRA	A288G	-	-	**4**	**2**	0.125	0.03	0.06	0.06	0.002	0.008
**M6151**	1994	FRA	A288G	-	-	**4**	**1**	0.06	0.125	0.5	0.25	0.001	0.008
**M6257**	2004	SWE	-	-	A2058G	0.25	0.5	0.03	0.015	1	0.5	**>16**	**>16**
**M6280**	1997	SWE	-	Pro62(59)→Ser C234T	-	0.5	0.25	0.03	0.03	0.125	0.06	0.0005	0.002
M6281*	2001	SWE	-	-	-	0.5	0.25	0.125	0.03	NT	NT	NT	NT
**M6282**	2003	JPN	-	-	-	**1**	0.5	0.03	0.03	0.5	0.5	0.001	0.004
**M6283**	2003	JPN	-	Ala69(66)→Thr	-	**1**	**1**	0.125	0.06	1	0.5	0.002	0.002
**M6284**	2003	JPN	-	-	-	**2**	**1**	0.06	0.06	0.125	0.125	0.001	0.004
**M6285**	1997	SWE	-	-	-	0.25	0.25	0.06	0.015	0.25	0.125	0.0005	0.002
**M6286**	2001	SWE	-	-	-	**2**	**1**	0.06	0.06	0.25	0.125	0.002	0.008
M6287*	2003	JPN	-	Asp87(84)→Tyr	-	**4**	**4**	0.5	0.125	0.25	0.125	0.002	0.004
M6312*	1994	FRA	-	-	-	**8**	**2**	0.125	0.125	NT	NT	NT	NT
**M6328**	1998	SWE	-	-	-	**8**	**2**	0.125	0.25	0.5	0.5	0.002	0.008
**M6489**	2007	SWE	Asp99(87)→Asn	Ser80(83)→Ile	A2059G	**>16**	**>16**	**16**	**1**	0.5	0.25	**>16**	**>16**
IMC-1*	2017	JPN	Gly93(81)→Cys	Ser80(83)→Ile	A2059G	**>16**	**>16**	**4**	**1**	1	0.125	**>16**	**>16**
OSSP35-2*	2017	JPN	Met95(83)→Ile	Ser80(83)→Ile	A2058G	**16**	**8**	**2**	0.25	0.5	0.125	**>16**	**>16**
JMPP4*	2017	JPN	-	-	-	**4**	**1**	0.25	0.06	0.25	0.125	0.002	0.03

NT, not tested.

*: For six strains, the MIC was determined by the cell-culture method

High MIC values ≥1 for fluoroquinolones or macrolides are shown in bold text. UK: United Kingdom, DK: Denmark, SWE: Sweden, FRA: France, JPN: Japan

M6489, IMC-1 and OSSP35-2 had elevated MICs for MXF with 16 mg/L, 4 mg/L and 2 mg/L, respectively. The MICs for STFX of these three strains were 1, 1 and 0.25 mg/L, respectively, and amino acid changes were found in both GyrA and ParC. The three strains had the same SNPs with amino-acid change Ser83→Ile in ParC (MXF: *p*<0.001, STFX: p = 0.012), but the amino acid changes in GyrA were different with Asp99→Asn, Gly93→Cys, and Met95→Ile, respectively.

MICs of CIP and LVX for all strains regardless of ParC or GyrA mutations were higher ranging from 0.5->16 mg/L and 0.25->16 mg/L, respectively. There was no significant difference of MICs of CIP or LVX between strains with or without SNPs in ParC (CIP: p = 0.621, LVX: p = 0.898). M2341, M6280 and M6283 had SNPs with ParC amino-acid changes of Pro62→Ser, Pro62→Ser and Ala69→Thr, respectively, but the MICs for MXF or STFX were low (range: 0.03–0.125 for MXF and 0.02–0.06 mg/L for STFX). M6287 had relatively high MIC for MXF (0.5 mg/L) and the amino-acid change Asp87→Tyr expected to result in elevated MXF MIC.

MICs for the tetracyclines such as DOX and MIN were almost similar in all strains (range: 0.06–1 mg/L for DOX and 0.06–0.5 mg/L for MIN).

### Temporal trend of macrolide and fluoroquinolone resistance mediating mutations in *M*. *genitalium* positive clinical samples collected in Japan, 2005–2017

Among the 148 *M*. *genitalium* positive urine specimens yielding readable sequences for all three targets, 84 were collected in 2005–2009 and 64 in 2010–2017 (Table 2). The prevalence of A2058G and A2059G SNPs in the 23S rRNA gene increased from 4.8% (4/84) in 2005–2009 to 42.2% (27/64) in 2010–2017 (*p*<0.001). The prevalence of any SNPs with amino acid changes in GyrA in each period was 0% (0/84) and 4.7% (3/64), respectively (p = 0.045). The prevalence of SNPs with amino acid changes in ParC increased from 22.6% (19/84) in 2005–2009 to 53.1% (34/64) in 2010–2017 (*p*<0.001). The percentage of *M*. *genitalium* positive urine specimens which had SNPs in both 23S rRNA and the QRDR in ParC remarkably increased from 0% (0/84) in 2005–2009 to 25.0% (16/64) in 2010–2017 (*p*<0.001) (Table 3). Especially, the percentage of specimens with macrolide-resistance and MXF-resistance with Ser83 (80)→Ile in ParC documented by in vitro MIC determination to be associated with resistance was 12.5% (8/64) in 2010–2017 (*p*<0.001).

**Table 2:** Prevalence of SNPs related to macrolide-resistance in domain V of the 23S rRNA gene and QRDRs of GyrA and ParC in 148 *M*. *genitalium* positive clinical specimens from patients’ first visit to medical facilities in Japan during 2006–2009 and 2010–2017.

Gene	SNP (*E*. *coli* numbering)	Amino acid change (*E*. *coli* numbering)	Numbers (%)		
			2005–2009 (n:84)	2010–2017 (n:64)	Total (n:148)
23S rRNA	A2071(2058)G		1 (1.2)	9 (14.1)	10 (6.8)
	A2072(2059)G		3 (3.6)	18 (28.1)	21 (14.2)
GyrA	G277T	Gly93 (81)→Cys	0	1 (1.6)	1 (0.6)
	G285T	Met95 (83)→Ile	0	2 (3.2)	2 (1.4)
	C201T		1 (1.2)	0	1 (0.6)
	T249G		0	1 (1.6)	1 (0.6)
	C267G		1 (1.2)	2 (3.2)	3 (2.0)
	G295A		1 (1.2)	0	1 (0.6)
ParC	C184T	Pro62 (59)→Ser	8 (9.5)	10 (15.6)	13 (8.8)
	G199T	Ala67 (64)→Ser	0	1 (1.6)	1 (0.6)
	G205A	Ala69 (66)→Thr	1 (1.2)	1 (1.6)	2 (1.4)
	G244A	Asp82 (79)→Asn	0	1 (1.6)	1 (0.6)
	A247G	Ser83(80)→Arg	0	1 (1.6)	1 (0.6)
	A248T	Ser83(80)→Ile	3 (3.6)	10 (15.6)	13 (8.8)
	A248A	Ser83(80)→Asn	5 (5.9)	6 (9.4)	11 (7.4)
	G259T	Asp87(84)→Tyr	1 (1.2)	1 (1.6)	2 (1.4)
	G259A	Asp87(84)→Asn	1 (1.2)	1 (1.6)	2 (1.4)
	G259C	Asp87(84)→His	0	1 (1.6)	1 (0.6)
	G359T	Ala119 (116)→Val	0	1 (1.6)	1 (0.6)
	C234T	-	4 (4.8)	4 (6.3)	8 (5.4)

**Table 3:** Prevalence of *M*. *genitalium* positive clinical specimens with SNPs with amino-acid change for both macrolide resistance in domain V of the 23S rRNA gene and fluoroquinolone-resistance mediating mutations in ParC in specimens from Japan.

SNPs with amino-acid change	2005–2009 (n:84)	2010–2017 (n:64)
23S rRNA (A2058G, A2059G) Plus all SNPs on ParC	0	16 (25.0%)
Pro62 (59)→Ser+Ala119 (116)→Val+C234T		1 (1.5%)
Ala69 (66)→Thr		1 (1.5%)
Ser83 (80)→Ile		8 (12.5%)
Ser83 (80)→Asn		5 (7.8%)
Asp87 (84)→Tyr		1 (1.5%)

## Discussion

Within the last 10 years, the treatment of *M*. *genitalium* infections has become increasingly difficult. Macrolides cannot be used as the first line treatment in many settings without testing for macrolide resistance mediating mutations as the prevalence of resistance is close to 50%.

*M*. *genitalium* is basically resistant to older fluoroquinolone such as CIP or LVX with high MICs for G37^T^ and other strains. On the other hand, MXF and STFX were highly active in these strains, and MIC of these agents for G37^T^ was eighth fold lower than that of LVX. However, MXF-resistant strains have emerged since 2007 where the first isolate M6489 was obtained from a patient failing treatment with high doses of DOX, AZM and other antimicrobials, as well as MXF [22]. However, the SNPs related with MXF-resistance have not been well documented due to the lack of cultured strains. Some studies have detected SNPs with amino-acid changes in the QRDR of ParC or GyrA in surveys based on sequencing directly from *M*. *genitalium* positive clinical specimens, and some have associated the mutations with treatment failure to MXF or STFX. Tagg *et al*. found nine SNPs with amino-acid changes in seven positions of ParC [11]. Deguchi *et al*. found Ser83→Ile or →Asn of ParC [13, 23]. Murray et al. showed Ser83→Ile or →Arg and Asp 87→Asn in ParC and in addition, some amino acid changes in GyrA [20]. Where the presence of SNPs has been correlated with treatment outcome, the serine in position 83 has been shown to be an important marker of MXF resistance. However, not all patients with *M*. *genitalium* infections with mutations such as Ser83→Ile or Asn, fail therapy with MXF and even less so with STFX [13, 20]. As shown in the present study, MICs for STFX appear to be less affected by ParC Ser 83 mutations than MXF explaining the higher cure rate of this compound. Particularly for STFX, amino acid changes in GyrA are probably needed as an additional factor for resistance. DNA loads in the specimens might also be related to treatment success with low loads being more prone to spontaneous clearance, and to false-negative follow-up tests [20]. Different mutations in the QRDR of GyrA or ParC have been seen in *M*. *genitalium* positive clinical specimens taken pre- and post-treatment. It is difficult to interpret whether the mutations were related to natural selection of mutations by the antimicrobial therapy or to a new infection from sexual contacts. Thus, associating mutations with treatment failure by detection of SNPs directly from clinical specimens has limitations, and the definitive proof needs to come from antimicrobial susceptibility testing of *M*. *genitalium* isolates. However, this is still difficult and very time consuming.

In the present study, we included three *M*. *genitalium* strains from patients with treatment–failure after MXF and/or STFX. M6489 was isolated from a patient treated with several antimicrobials including high-dose MXF. IMC-1 was the first strain with a high MIC to MXF from Japan, and this patient was also treated with several antimicrobials including AZM, MXF and STFX twice with 200 mg/day for 7 days. OSSP35-2 was a unique strain. This strain was isolated from a patient treated with AZM and STFX. *M*. *genitalium* was eradicated by STFX but not by AZM. Therefore, this strain is thought to be clinically STFX-sensitive and AZM-resistant. OSSP35-2 had MIC >16 mg/L for both AZM and CLR and had the A2058G SNP in the 23S *r*RNA gene and thus, confirmed as macrolide-resistant. The MIC of MXF was 2 mg/L and STFX was 0.25 mg/L, which is thought to be susceptible to STFX, but resistant to MXF. The same SNPs were observed before and after treatment in the clinical specimens suggesting transmitted resistance.

Despite having the same ParC Ser83→Ile mutation, the MICs of MXF in M6489, IMC-1 and OSSP35-2 were different with 16, 4 and 2 mg/L, respectively. However, the three strains had three different GyrA mutations. The SNPs resulting in amino acid changes Asp99→Asn (M6489) and Met95→Ile (OSSP35-2) were previously found by Murray *et al*. [20]. In this report, the microbiological outcomes of MXF in patients who had *M*. *genitalium* positive specimens with both Ser83→Ile and Met95→Ile were mixed with two of four cured and two with persistence. The MIC of 2 mg/L of MXF for OSSP35-2 thought to be just above the CLSI susceptible breakpoint for pneumococci for MXF, thus, it may be reasonable that a proportion of patients may experience cure. The patient from whom IMC-1 was isolated was treated with MXF 400mg/day for 38 days and the strain was isolated after the treatment and had an MIC of 4 mg/L, this is clearly above the breakpoint for susceptibility. It carried a GyrA Gly93→Cys mutation not previously described, but within the core of the QRDR.

Based on our findings, we propose that the Ser83→Ile mutation in ParC is closely related to MXF resistance (*p*<0.001), but that the level of MIC may be modified by mutations in GyrA. Mutations in GyrA alone does, however, not appear to result in MXF resistance. Furthermore, ParC mutations outside of the core QRDR (position 83–87) such as the common Pro62→Ser mutation does not appear to influence cure rates after MXF. The mutation on Asp87 in ParC is a possible site related to MXF-resistance. In this study, MXF MIC was relatively high at 0.5 mg/L and a mutation on this amino-acid has been reported in other studies. Isolation of strains with mutations in Asp87 are needed to document the importance of these mutations.

STFX has a unique antimicrobial activity and can eradicate CIP-resistant *Neisseria gonorrhoeae* strains with amino-acid mutations in the QRDR of GyrA and ParC [32]. In contrast, such strains cannot be eradicated with MXF. STFX is also effective against quinolone-resistant *Escherichia coli* strains. Among 193 *E*. *coli* strains that had ≥3 mutations in the QRDR of GyrA or ParC genes, 66% strains were susceptible to STFX [33]. Even though STFX appears to be more potent than MXF for *M*. *genitalium* strains with ParC Ser 83 mutations, the pharmacokinetic-pharmacodynamic calculated breakpoint for pneumococci is also lower than that of MXF with 0.125 mg/L for the 200 mg daily dose compared to 0.5 mg/L for MXF 400 mg daily [34]. Thus, even the OSSP35-2 strain with an STFX MIC of 0.25 mg/L may be considered intermediate susceptible although treatment was successful.

The prevalence of *M*. *genitalium* positive specimens with macrolide- or fluoroquinolone-resistance related SNPs has been increasing in Japan. If strains with A2058G or A2059G in the 23S rRNA and SNPs with amino-acid change in Ser83 or Asp87 of ParC are considered multi-drug resistant, the prevalence of multi-drug resistance might be 21.8% in 2010–2017 in Japan. If SNP on ParC is strictly limited to Ser83→Ile, the multi-drug resistance rate might be 12.5%. These figures are in line with other studies from Japan [13, 24]. The current first-line therapy for non-gonococcal urethritis in Japan is AZM, and STFX is used as the second-line treatment if AZM fails. However, we have no therapy for treatment failure after AZM and STFX, and the treatment of *M*. *genitalium* infections is becoming increasingly difficult. Consequently, new antimicrobials are urgently needed, and isolation of contemporary *M*. *genitalium* strains is important to increase our understanding of the relation between SNPs and elevated MICs and for evaluation of new therapy.
